# The Impact of Limited Access to Dental Care on Emergency Room Service Utilization: A Study of Primary Healthcare in a Rural Inland Region of Portugal

**DOI:** 10.3390/dj14070411

**Published:** 2026-07-06

**Authors:** Alexandra Prada, Ana Galvão, Matilde Monteiro-Soares, Cláudia Camila Dias

**Affiliations:** 1 ULS—Unidade Local de Saúde do Nordeste, 5301-852 Bragança, Portugal; 2 Faculty of Medicine, University of Porto, 4200-319 Porto, Portugal; 3 Research Centre for Active Living and Wellbeing (LiveWell), Instituto Politécnico de Bragança (IPB), 5300-253 Bragança, Portugal; 4 Department of Social, Life and Public Health Sciences, Instituto Politécnico de Bragança, 5300-253 Bragança, Portugal; 5 CINTESIS@RISE—Center for Health Technology and Services Research, Faculty of Medicine, University of Porto, 4200-319 Porto, Portugal; 6 Portuguese Red Cross Health School Lisbon, 1300-125 Lisbon, Portugal; 7 Cross I&D, 1000-1990 Lisbon, Portugal; 8 Knowledge Management Unit, Faculty of Medicine, University of Porto, 4200-319 Porto, Portugal; 9 RISE-Health, Department of Community Medicine, Information and Health Decision Sciences (MEDCIDS), Faculty of Medicine, University of Porto, 4200-319 Porto, Portugal

**Keywords:** oral health, dental pain, emergency department, rural health, DMFT index, access to dental care, Portugal

## Abstract

**Background/Objectives:** This cross-sectional observational study investigated factors associated with emergency room (ER) utilization for dental pain in a rural inland region of Portugal. The main objective was to examine the relationship between access to dental care, sociodemographic characteristics, oral health behaviors, and clinical outcomes with the use of emergency room services for dental problems. **Methods:** The study sample comprised 423 participants from the districts of Bragança and Vinhais, in Trás-os-Montes, aged 4 to 90 years, who attended their first dental medicine consultation. Participants completed a structured questionnaire addressing sociodemographic characteristics, general health, oral health behaviors, and dental prosthetic use, and underwent oral examination for assessment of the Decayed, Missing, and Filled Teeth (DMFT) index. Associations with reported ER utilization due to toothache were analyzed using Fisher’s exact test and the Mann–Whitney U test. **Results:** Overall, 28.4% of participants reported having visited the ER due to dental pain, and most cases were managed with medication followed by discharge. ER utilization was significantly associated with behavioral risk factors such as smoking, as well as poorer oral hygiene practices, including less frequent tooth brushing. In addition, participants who sought ER care presented higher DMFT scores, indicating a greater burden of untreated dental decay and tooth loss. **Conclusions:** These findings suggest that limited preventive dental care and unfavorable oral health behaviors are associated with to avoidable ER visits for dental pain in rural settings. This study reinforces the need to strengthen access to preventive oral health services and to advance the integration of dental care into Portugal’s National Health Service (SNS), particularly in underserved inland regions.

## 1. Introduction

Oral health is an essential component of overall health and well-being, with effects that extend beyond the mouth and are associated with systemic conditions such as cardiovascular disease, diabetes, and respiratory illnesses [1,2]. Regular dental care is therefore important for preventing oral diseases and reducing broader health risks. However, access to dental care remains a significant challenge, particularly for vulnerable populations, leading to untreated oral diseases and increased demand for emergency dental services [3,4].

In Portugal, dental care is predominantly delivered through the private sector, creating barriers to access, especially for low-income and rural populations. National statistics indicate that approximately 9.9% of individuals aged 16 years or older reported unmet dental care needs in 2024 due to financial constraints [5]. Surveys also report that many adults have poor dentition, out-of-pocket payments are common, and public dental services via the National Health Service are rarely used [6]. According to the Barómetro da Saúde Oral 2024, 30% of Portuguese adults avoid dental care due to cost, nearly one quarter seek care only in emergencies, and public support for state participation in dental care financing is high [7]. These trends mirror broader global concerns, where out-of-pocket financing limits access and exacerbates inequalities, even in high-income countries [8].

The reliance on emergency dental services places a burden on healthcare systems, as emergency departments often manage preventable cases, such as acute pain, infections, and trauma, which could be addressed through regular dental care [9]. The lack of preventive care, poor oral hygiene, financial constraints, fear of dental visits, and limited awareness of preventive care may contribute to delayed treatment and emergency-based patterns of care [10]. International studies show that early preventive visits are linked to better oral health outcomes and reduced emergency care utilization [11,12]. Preventive dental care, including regular check-ups, early interventions, and oral hygiene education, also reduces preventable conditions such as cavities and gum disease [13] and may reduce the economic burden on emergency departments, as early treatment is generally more cost-effective than urgent care [14].

Comprehensive studies in Portugal examining the relationship between dental care access and emergency service utilization remain limited. Previous research has explored oral health behaviors and socioeconomic inequalities [15,16,17], but few studies directly link these factors to emergency service use. This study aims to examine the relationship between access to dental care and reported emergency service utilization due to dental pain.

## 2. Material and Methods

### 2.1. Study Design

This was a cross-sectional, observational, and analytical study conducted within primary healthcare settings in a rural inland region of Portugal.

### 2.2. Study Participants

The study sample comprised individuals of all ages (*n* = 423) who attended primary healthcare units in the rural inland region of Portugal (Bragança and Vinhais) from November 2024 to February 2025 and provided informed consent to participate. During the data collection period, a total of 1001 dental appointments were conducted across the participating primary healthcare units. Of these, 423 corresponded to first dental consultations and were therefore eligible for inclusion; the remaining 578 were follow-up appointments and were excluded from the study. Participants were patients referred to the dental medicine appointment by their family physician, within the primary healthcare setting. The questionnaire was completed by the dentist during the participants’ first dental appointment at the healthcare center, as part of the clinical anamnesis, to better understand each patient’s oral health status, behaviors, and dental care needs. To minimize interviewer bias, the questionnaire was administered in a standardized manner by the same dentist, using the same sequence of questions and neutral wording for all participants. The interviewer avoided suggesting or interpreting answers during data collection, recording participants’ responses as provided. Although the questionnaire was completed during the clinical appointment, the data used for the study were anonymized and analyzed independently, ensuring that participants could not be identified in the research database.

The sample comprised residents from both urban and rural areas and included participants from diverse socioeconomic backgrounds. Exclusion criteria were participants unable to complete the questionnaire due to cognitive or language barriers. However, no individuals were excluded during the data collection period.

### 2.3. Questionnaire

Data were collected using a structured questionnaire, which was divided into four main sections: sociodemographic information, general health, oral health, and dental prosthetics. The questionnaire was developed based on previously published scientific studies addressing oral health behaviors, access to dental care, and emergency service utilization [3,9,17], and was further adapted to incorporate clinically relevant variables specific to the objectives of the present study. To ensure content adequacy and clarity, is was reviewed prior to implementation by healthcare professionals (colleagues from the Unidade Local de Saúde do Nordeste) with experience in oral health and primary care. The complete questionnaire (File S1) is available in the Supplementary Materials.

The first section, on sociodemographic information, comprised questions gathering basic demographic data, including gender; age or date of birth; residence (urban or rural); educational level (ranging from mandatory education to doctorate); employment status (which included categories such as employed, unemployed, student, self-employed, retired, and permanently disabled); marital status; and household composition, including the number of individuals living in the participant’s household.

The second section, on general health, contained questions collecting information about the participants’ health status. This included questions regarding current medication use, with a focus on common conditions such as hypertension, diabetes, asthma, depression, and others. Additionally, participants were asked about any allergies, particularly to medications, and their medical history, including chronic diseases, surgeries, and family history of conditions like cancer. This section also covered lifestyle habits, asking about smoking, alcohol consumption, and physical activity levels.

The third section, oral health, addressed various aspects of the participants’ oral health. Participants were asked about the reasons for their dental visits, such as routine check-ups, dental pain, or prosthetic placements. The questionnaire also included questions about oral hygiene habits, including the frequency of tooth brushing, use of dental floss, and the type of toothbrush used. Dental history was explored through questions regarding the participant’s first dental visit, their last dental visit, and the frequency of dental visits within the last 12 months. Symptoms such as dry mouth and teeth grinding (bruxism) were also investigated. Finally, participants were asked if they had ever sought emergency dental services for pain and the treatments they had received during such visits.

The fourth section, dental prosthetics, collected information on the use of dental prosthetics, including whether participants currently used any type of prosthesis, the type of prosthesis (e.g., removable or fixed), and the frequency of use. Participants were also asked about their satisfaction with their prosthesis, any discomfort or pain experienced, and whether they were able to eat normally while using it. Questions regarding the maintenance of prosthetics, such as whether participants cleaned them and how often, were also included. The section also covered the duration of prosthesis use, asking participants how long they had been using their current prosthesis.

### 2.4. Data Collection Procedure

Data were collected through structured interviewer-administered questionnaires. The questionnaire was completed by the dentist during each participant’s first dental appointment, as part of the clinical anamnesis. The dentist explained the purpose of the study to each participant prior to data collection and recorded responses as provided. Participants were informed about the study’s objectives and procedures before completing the questionnaire.

Participants were allowed to complete the questionnaire during their visit, which took approximately 15 to 20 min. Once completed, the questionnaires were returned to the healthcare staff for further processing.

### 2.5. DMFT Index Assessment

Oral health status was assessed through clinical examination using a structured dental chart that recorded the condition of each tooth according to predefined diagnostic codes (see the questionnaire at the Supplementary Materials). Dental caries experience was measured using the Decayed, Missing, and Filled Teeth (DMFT) index for both permanent and primary dentition, in accordance with the criteria established by the World Health Organization [18]. Teeth were classified as decayed (cavitated lesions), missing due to caries, or filled, based on clinical observation. Teeth lost for reasons other than caries were recorded separately and not included in the DMFT calculation. The total DMFT score was calculated as the sum of its components (D + M + F), with higher scores indicating a greater cumulative burden of dental caries.

### 2.6. Statistical Analysis

Descriptive statistics were first applied to summarize the data, including frequencies and percentages for categorical variables and means and standard deviations or medians and interquartile ranges for continuous variables.

To examine the relationships between independent variables, namely, oral hygiene habits, alcohol consumption, smoking habits, presence of comorbidities, dental visits (within the last 12 months), use of dental prosthetics, and stomach problems, and the dependent variable, namely, the use of emergency services for dental pain, Chi-square tests (Pearson Chi-square or Fisher’s exact test for 2 × 2 contingency tables) were used.

To assess differences in age at first dental visit and components of the DMFT index based on whether participants had utilized emergency services for dental pain, the Shapiro–Wilk test was used to assess normality, and the Mann–Whitney U test was applied for group comparisons.

In addition, a multivariable logistic regression analysis was performed to assess adjusted associations with emergency room utilization due to dental pain. Emergency room utilization due to dental pain was included as the dependent binary variable. Variables with clinical relevance and/or statistically significant associations in the bivariate analyses were considered for independent variables, namely age, DMFT index for permanent teeth, current smoking, alcohol consumption an medication use Results were expressed as adjusted odds ratios (aORs) with 95% confidence intervals (95% CIs).

The significance level for all statistical tests was set at *p* < 0.05.

Statistical analysis was performed using IBM SPSS Statistics 30.0 (Chicago, IL, USA). Additional descriptive results on general health, health behaviors, and dental prosthesis use are provided in Tables S1–S3 (Supplementary Materials). Sensitivity analysis results are presented in Table S4 (Supplementary Materials).

### 2.7. Ethical Considerations

The study was conducted in full compliance with ethical standards, as outlined in the Declaration of Helsinki. The research protocol was reviewed and approved by the Ethics Committee of the *Unidade Local de Saúde do Nordeste* (approval number: 65/2024).

## 3. Results

### 3.1. Sociodemographic Characteristics of the Sample

**Figure 1:**
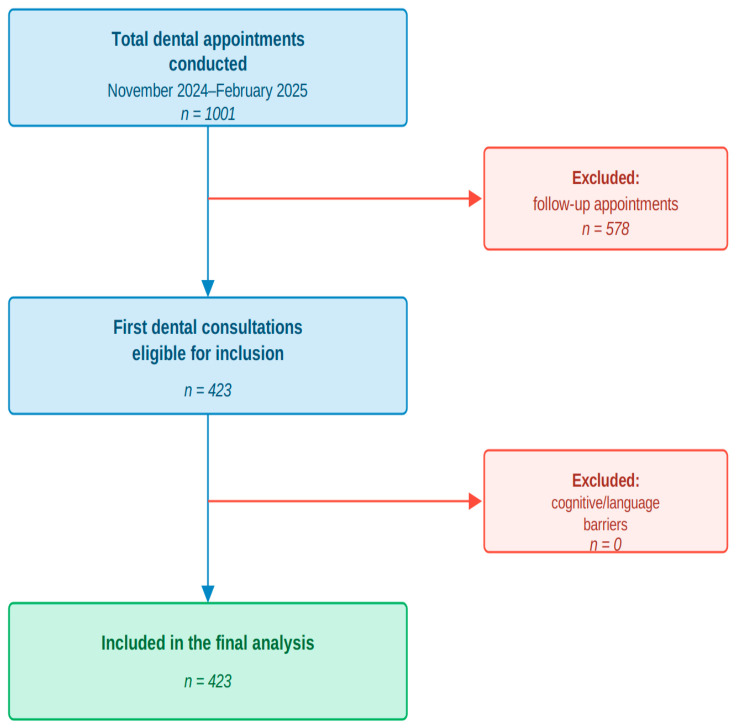
Recruitment flow diagram illustrating participant selection during the data collection period (November 2024–February 2025).

The participant selection process is illustrated in Figure 1. During the data collection period (November 2024–February 2025), a total of 1001 dental appointments were conducted. Of these, 423 corresponded to first dental consultations and were included in the analysis; the remaining 578 were follow-up appointments and were excluded. No participants were excluded due to cognitive or language barriers. A total of 423 participants completed the questionnaire, with ages ranging from 4 to 90 (mean age: 32.2 ± 26.4 years). The sample was predominantly male (57.7%), with 54.8% living in urban areas (see Table 1). Regarding education, 34.3% of participants had completed only primary education, while a smaller proportion had attained secondary education (14.7%) or higher education. Only 5.0% of the sample held a bachelor’s degree, indicating a relatively low level of higher education. Employment data showed that 46.1% of participants were students, 17.7% were retired, and 14.2% were unemployed. A smaller percentage (1.7%) were self-employed, while 11.1% worked in either the private or the public sector. Marital status distribution revealed that most participants were single (66.7%) (Table 1).

Regarding institutional living arrangements, 8.0% of participants resided in institutions, the majority of whom lived in orphanages (64.7%).

Concerning household composition and healthcare access, most participants lived in households of two to four people, with 27.4% in two-person households and 18.7% in four-person households. The majority (93.4%) had a family doctor, and 15.1% had supplementary health coverage, such as private insurance or a health subsidy. Among those with additional coverage, private health insurance was the most prevalent (51.6%), followed by the ADSE system (42.2%) (Table 1).

Additional descriptive data on general health characteristics, including medication use, allergies, stomach problems, surgical history, and family history of cancer, are presented in Table S1 (Supplementary Materials).

### 3.2. Oral Health Behaviors and Practices

Regarding oral health habits, most participants reported brushing their teeth or cleaning their dentures at least once a day, with 40.4% brushing once daily (Table 2). When it comes to the type of toothbrush used, 82.4% of participants used a manual toothbrush, while 5.9% used an electric toothbrush. Additionally, 11.7% used both manual and electric toothbrushes.

The use of toothpaste was widespread, with 92.4% of participants reporting that they used toothpaste. However, the use of dental floss was much less common, with only 7.1% of participants using it regularly. Similarly, the use of mouthwash or oral rinses was reported by 19.6% of participants, suggesting that supplementary oral hygiene practices were not widely adopted.

In terms of symptoms related to oral health, 25.8% of the participants reported experiencing dry mouth (Table 2). Among those affected, 53.2% attempted to compensate for this condition by drinking more water. Additionally, 28.6% of the participants reported grinding their teeth, and 14.9% frequently bit their lips or cheeks.

Regarding the reason for the current dental visit, 55.6% of the participants sought dental care for the first time due to pain, highlighting the reactive nature of dental care utilization in the sample. Only 7.1% attended the dentist for routine check-ups, and 11.8% attended through a dental check-up program. Moreover, 67.4% of the participants had not visited the dentist in the past year, with the main reasons for not visiting being cost-related (66.3%) and perceived lack of need for dental care (28.4%). Among those who did visit a dentist in the past year, most sought treatment for pain (33.3%) or for ongoing treatments (55.1%) (Table 3).

Additional descriptive data on health behaviors and risk factors, including physical activity, alcohol consumption, and smoking habits, are presented in Table S2 (Supplementary Materials).

### 3.3. Emergency Service Utilization

When asked about utilization of hospital emergency services, 28.4% of participants (*n* = 120) reported seeking care due to dental pain (Table 3). Among those who accessed emergency services, the majority (88.3%) were treated with medication and discharged, while 11.7% were referred to an oral specialist for further care.

### 3.4. Association Between Behavioral and Clinical Factors and Utilization of Emergency Room Services

Several behavioral and clinical factors were found to be significantly associated with the use of emergency room services for dental pain. An association was observed with smoking and alcohol consumption. Participants who never smoked reported lower use of emergency room services (20.9%) compared with current smokers (50.6%) and former smokers (50.0%) (*p* < 0.001; Table 4). Similarly, ER service use was lower among non-drinkers (22.9%) compared with those who drank both during and outside meals (52.8%). This association should be interpreted with caution, given the potential confounding effect of age associated with the inclusion of participants under 18 years of age. A sensitivity analysis restricted to adult participants (aged ≥ 18 years) was performed to assess the potential influence of age-related confounding; the results are presented in Supplementary Table S4. The analysis showed a change in the magnitude of the association, suggesting the presence of confounding and reinforcing the need for adjusted analyses to confirm these findings. The analysis restricted to the adult population shows a change in the magnitude of the association, suggesting the presence of confounding and the need for adjusted analyses to confirm these findings (*p* < 0.001; Table 4).

Additionally, the presence of comorbidities such as hypertension, diabetes, and heart disease was linked to a higher likelihood of utilizing emergency room services. Participants with comorbidities reported greater ER service use (47.5%) compared with those without comorbidities (30.0%) (*p* = 0.017; Table 4).

The use of dental prostheses was significantly associated with emergency room utilization. Participants using dental prostheses reported higher emergency room use than those not using prostheses (51.1% vs. 25.5%; *p* = 0.029; Table 4). Additional descriptive data on dental prosthesis use and related experiences are presented in Table S3.

Dry mouth and gastrointestinal issues were also associated with emergency room utilization. Emergency room use was higher among participants with dry mouth than among those without this symptom (48.6% vs. 21.3%; *p* < 0.001), and among those reporting stomach problems (49.5%; *p* < 0.001; Table 4).

Oral hygiene habits were significantly associated with emergency room utilization. Participants who did not brush their teeth daily reported higher emergency room use than those brushing twice or three times daily (53.8% vs. 15.9% and 13.6%, respectively; *p* < 0.001; Table 4). Similarly, participants without a personal toothbrush reported higher emergency room use than manual or electric toothbrush users (78.6% vs. 30.6% and 8.3%, respectively; *p* < 0.001). Mouthwash use was also associated with emergency room utilization (*p* = 0.049; Table 4).

Recent access to dental care was significantly associated with emergency room utilization. Participants who had not attended a dental appointment in the previous 12 months reported higher emergency room use than those who had attended a consultation (33.7% vs. 17.4%; *p* = 0.037; Table 4).

A sensitivity analysis restricted to adult participants (aged ≥ 18 years) is presented in Supplementary Table S4. In this subgroup, statistically significant associations with emergency room utilization were observed for dry mouth (*p* = 0.027), stomach issues (*p* = 0.047), toothpaste use (*p* < 0.001), mouthwash use (*p* < 0.001), and having had a dental appointment in the last 12 months (*p* < 0.001). In contrast, associations previously observed in the full sample for smoking (*p* = 0.198), alcohol consumption (*p* = 0.056), presence of comorbidities (*p* = 0.434), use of dental prosthesis (*p* = 0.139), and tooth brushing frequency (*p* = 0.064) did not reach statistical significance in the adult-only analysis, suggesting that some of these associations may be influenced by the inclusion of younger participants and should be interpreted with caution.

### 3.5. DMFT Index and Oral Health Outcomes

The DMFT index was used to assess the overall oral health status of participants. The results presented in Table 5 indicate generally poor and highly heterogeneous dental status within the sample. The DMFT index for permanent teeth shows a median of 5 and a wide range (0–32), with an interquartile range from 0 to 15, highlighting substantial variability and a skewed distribution. In contrast, the DMFT for deciduous teeth is minimal, with a median of 0 and values concentrated at zero (P25 = 0; P75 = 0), suggesting little involvement of primary dentition. Among the components, decayed and missing teeth contribute most to the overall DMFT, both presenting higher upper quartile values (P75 = 6); whereas, filled teeth remain low (median = 0; P75 = 1), indicating limited restorative care. Additionally, the Shapiro–Wilk test is significant for all variables (*p* < 0.001), confirming non-normal distributions, consistent with the asymmetry, wide ranges, and clustering of observations at zero.

The results in Table 6 reveal significant differences between individuals with and without a dental pain episode requiring emergency room services. Those who reported emergency care present markedly worse oral health, with substantially higher median values for DMFT in permanent teeth (14 vs. 1), as well as higher levels of decayed teeth (median 4 vs. 1) and missing teeth (median 6 vs. 0), all with statistically significant differences (*p* < 0.001). In contrast, no meaningful differences are observed for filled teeth (*p* = 0.187), suggesting that restorative care does not distinguish between the groups. For deciduous teeth, although both groups show medians of zero, the difference remains statistically significant (*p* < 0.001), likely reflecting distributional differences beyond central tendency. Additionally, individuals who required emergency room services had a notably older age at first dental visit (median 17 vs. 6; *p* < 0.001), indicating delayed access to dental care. Overall, these findings suggest that poorer oral health status and delayed engagement with dental services are strongly associated with the occurrence of dental pain episodes requiring emergency room services.

### 3.6. Multivariable Logistic Regression Analysis of Emergency Room Utilization Due to Dental Pain

A multivariable logistic regression analysis in Table 7 was performed to assess factors associated with emergency room utilization for dental pain. The model included age, DMFT index for permanent teeth, current smoking, alcohol consumption, and presence of reported comorbidities medication use. The overall model was statistically significant, χ^2^(5) = 68.536; *p* < 0.001, with a Nagelkerke R^2^ of 0.215, suggesting that approximately 21.5% of the variability in emergency room utilization due to dental pain was explained by the variables included in the model. The overall classification accuracy was 73.5%.

The DMFT index for permanent teeth remained significantly associated with emergency room utilization due to dental pain. Each one-point increase in the DMFT index was associated with a 7.1% increase in the odds of emergency room utilization due to dental pain (aOR = 1.071; 95% CI: 1.036–1.107; *p* < 0.001). Current smoking was also significantly associated with the outcome, with current smokers showing approximately 2.47 times higher odds than non-current smokers (aOR = 2.466; 95% CI: 1.350–4.506; *p* = 0.003). Alcohol consumption, medication use, and age were not significantly associated with emergency room utilization after adjustment.

## 4. Discussion

This study provides a comprehensive examination of factors associated with ER service utilization for dental pain in a rural inland Portuguese population, focusing on sociodemographic factors, oral health behaviors, and clinical outcomes measured by the DMFT index. Although previous studies in Portugal have explored oral health behaviors and socioeconomic inequalities [15,16,17], few have directly examined how access to dental care, behavioral factors, and clinical oral health status relate to the use of emergency services for dental pain. In this sense, the present study adds evidence from an underserved rural setting and reinforces the relevance of oral diseases as a major global public health challenge [19].

One of the main findings of this study was the association between behavioral factors, particularly smoking and alcohol consumption, and ER utilization for dental pain. These findings align with previous studies in Portugal and internationally, which have shown that poor lifestyle habits contribute to the progression of oral conditions requiring urgent care [20,21]. Smoking is well established as a risk factor for periodontal disease, tooth decay, and oral cancer [22]. Similarly, alcohol consumption can exacerbate oral health problems by promoting dry mouth, increasing the risk of tooth decay, and damaging oral tissues [23].

Poor oral hygiene practices are widely recognized as significant predictors of dental decay and periodontal disease, both of which are leading causes of dental pain [24]. The results mirror findings from [25], who reported that individuals with poor oral hygiene are more likely to seek emergency care for problems that could be prevented with appropriate self-care. This highlights the importance of oral health education and preventive interventions, especially among vulnerable populations.

In addition, 28.4% of participants reported having attended an emergency room due to toothache. Although this item does not allow conclusions about the main reasons for emergency room visits in general, it suggests that dental pain may lead some individuals to seek urgent care rather than preventive or routine dental services. This finding may reflect delayed help-seeking behavior and potential gaps in timely access to dental care.

Another relevant finding was the association between dental prosthetics and emergency care use. This may be related to dissatisfaction, discomfort, pain, or inadequate prosthetic adaptation, which can increase the need for urgent care. Inadequate prosthetic care, such as ill-fitting dentures or improper maintenance, can lead to functional difficulties, discomfort, and poorer oral health-related quality of life [26,27]. In addition, inadequate denture hygiene and irregular follow-up have been associated with oral mucosal lesions and poorer denture care habits, which may further increase treatment needs [28]. These findings highlight the importance of follow-up care and patient education regarding prosthetic maintenance and proper fitting.

This study also analyzed the DMFT index, which showed that participants with higher scores, indicating more severe dental decay and tooth loss, were more likely to seek emergency care for dental pain. This finding is consistent with studies linking poor oral health, as measured by the DMFT index, to increased reliance on emergency dental services. Some studies found that children [29,30] with higher DMFT scores, particularly those with untreated caries and tooth loss, are more likely to use emergency services. Taken together, these findings suggest that ER utilization for dental pain is shaped by a combination of behavioral factors, clinical conditions, and limited engagement with preventive dental care.

The adjusted logistic regression analysis showed that the DMFT index for permanent teeth and current smoking remained associated with emergency room utilization due to dental pain after adjustment for age and other covariates. Higher DMFT scores may reflect a greater cumulative burden of oral disease, which can increase the likelihood of urgent care-seeking due to dental pain. Current smoking was also associated with higher odds of emergency room utilization, supporting its role as a behavioral factor related to poorer oral health outcomes, including periodontal disease, dental caries, and oral cancer [20,22]. Alcohol consumption, medication use, and age were not significantly associated with emergency room utilization after adjustment.

One of the most important implications of this study is the need to strengthen preventive dental care and improve access to oral health services within the SNS. The results suggest that some individuals may not receive timely preventive care, allowing dental problems to progress until urgent intervention is needed. Expanding dental coverage within the SNS could support access to regular check-ups, oral hygiene education, and early interventions before dental conditions become severe, in alignment with global strategies advocating for the integration of oral health into universal health coverage frameworks [31].

International models have demonstrated the effectiveness of including dental care within broader public health systems [32]. For example, publicly funded oral health services within national health systems may improve access to preventive and emergency dental care, while comparative analyses suggest that stronger public dental coverage can help meet the needs of populations with poorer oral health and reduce disparities in utilization. These international examples support global public health guidance emphasizing the integration of oral health into national health policies as a step towards universal health coverage.

In Portugal, the lack of universal dental coverage remains a significant barrier to equitable oral health care. Many individuals, particularly those from lower socioeconomic backgrounds or rural areas, face financial and geographic barriers to accessing regular dental care. This study underscores the need for policy reforms to improve access to essential dental services, regardless of income or geographic location. Expanding dental coverage within the SNS may contribute to reducing oral health inequities and preventing avoidable emergency care arising from preventable conditions.

While this study offers valuable insights, it also has limitations. The study relied on self-reported data, which may introduce bias, particularly regarding the frequency of dental visits, symptoms, and emergency service use. The sample was geographically limited to a rural inland population in Portugal and may not fully represent the broader population. Future studies should include larger and more diverse samples to improve the generalizability of the findings.

Moreover, longitudinal research is needed to better understand the causal relationships between preventive care, oral health behaviors, and emergency care utilization. Longitudinal studies could also assess the impact of integrating dental care into the SNS on emergency service use visits, providing important evidence on the effectiveness of such policy reforms.

## 5. Conclusions

Emergency room utilization due to dental pain in rural inland Portugal appears to be associated with poorer oral health status and selected behavioral factors. In this study, participants with higher DMFT scores, reflecting a greater burden of dental caries, missing teeth, and previous restorative treatment, were more likely to report emergency room use due to dental pain. Current smoking also remained associated with emergency room utilization in the adjusted analysis, suggesting that smoking may contribute to poorer oral health outcomes and a greater need for urgent dental care. Although alcohol consumption and inadequate oral hygiene practices were associated with emergency room utilization in the bivariate analyses, these associations should be interpreted cautiously, as they did not remain significant after adjustment and require further investigation in larger studies.

These findings suggest that preventable oral conditions may contribute substantially to emergency room use for dental pain, particularly in rural and socioeconomically vulnerable populations. Individuals with untreated dental disease, tooth loss, or limited access to routine dental care may delay treatment until symptoms become severe, leading to reliance on urgent services. This pattern highlights potential gaps in preventive oral healthcare and reinforces the importance of early detection, regular dental follow-up, and timely treatment of oral diseases.

From a public health perspective, reducing avoidable emergency room visits due to dental pain requires strengthening preventive strategies and improving access to dental care, especially in rural areas. Regular oral health screening, oral hygiene education, smoking-related counseling, and earlier referral to dental services may help reduce the burden of untreated oral disease. Improving access to routine and preventive dental care within Portugal’s National Health Service may also contribute to reducing oral health inequalities and promoting more equitable access to care. Further research using larger and more representative samples is needed to confirm these findings and to evaluate the effectiveness of preventive and policy interventions aimed at reducing emergency room utilization for dental pain.

## Figures and Tables

**Table 1:** Sociodemographic characterization of the sample (*n* = 423, unless otherwise specified).

	*n*	%
**Gender**		
Male	244	57.7
Female	179	42.3
Age (years), mean (standard deviation)	32.2	(26.4)
**Residence**		
Rural	191	45.2
Urban	232	54.8
**Education level**		
No education	17	4.0
Kindergarten	53	12.5
Primary education (First cycle)	145	34.3
Lower secondary (Second cycle)	56	13.2
Upper secondary (Second cycle)	69	16.3
High school (Secondary education)	62	14.7
Bachelor’s degree	21	5.0
**Employment status**		
Unemployed	60	14.2
Student	195	46.1
Self-employed	7	1.7
Employee	47	11.1
Retired	75	17.7
Permanently unable to work	39	9.2
**Marital status**		
Single	282	66.7
Married	79	18.7
Divorced	24	5.7
Widowed	24	5.7
Other	14	3.3
**Living in an institution**		
No	389	92.0
Yes	34	8.0
**Type of institution** (*n* = 34)		
Nursing home	5	14.7
Orphanage	22	64.7
Association	7	20.6
**Household size**		
1	39	9.2
2	116	27.4
3	76	18.0
4	79	18.7
5	32	7.6
6	33	7.8
7	14	3.3
Living in a community or institution	34	8.0
**Family doctor**		
No	28	6.6
Yes	395	93.4
**Health insurance**		
No	359	84.9
Yes	33	7.8
Subsystem	31	7.3
**Type of health subsystem** (*n* = 64)		
ADSE	27	42.2
SAD-PSP	2	3.1
SAMS	2	3.1
Private health insurance	33	51.6

**Table 2:** Oral health and hygiene habits (*n* = 423, unless otherwise specified).

	*n*	%
**Brushes teeth and/or prosthesis daily**		
No	39	9.2%
Yes	384	90.8%
**If yes, how many times do you brush your teeth per day?** (*n* = 384)		
Once a day or occasionally	155	40.4%
Once a day	94	24.5%
Twice a day	113	29.4%
Three times a day	22	5.7%
**Has a personal toothbrush**		
No	14	3.3%
Yes	409	93.7%
**If yes, which type of toothbrush do you use?** (*n* = 409)		
Manual	337	82.4%
Electric	24	5.9%
Both above	48	11.7%
**Uses toothpaste?**		
Does not use	32	7.6%
Yes	391	92.4%
**Uses dental floss regularly?**		
Does not use	393	92.9%
Yes	30	7.1%
**Uses mouthwash?**		
Does not use	340	80.4%
Yes	83	19.6%
**Has a “dry mouth”?**		
No	314	74.2%
Yes	109	25.8%
**If yes, compensates by drinking more water?** (*n* = 109)		
No	12	11.0%
Occasionally	39	35.8%
Yes	58	53.2%
**Habitually grinds teeth?**		
Does not	302	71.4%
Yes	121	28.6%
**Frequently bites own lips/cheeks?**		
Does not	360	85.1%
Yes	63	14.9%

**Table 3:** Use of health and emergency room services (*n* = 423, unless otherwise specified).

	*n*	%
Reason for requesting the dental medicine appointment at the healthcare center (*n* = 416)		
Pain	205	49.3%
Routine	78	18.4%
Placement of prosthesis	21	5.0%
Screening at school	107	25.3%
Other	5	1.2%
Waiting time for primary healthcare consultation		
Around 15 days	119	28.1%
Around 30 days	100	23.6%
Around 50 days	5	1.2%
Around 60 days	57	13.5%
Around 70 days	2	0.5%
Around 80 days	5	1.2%
Around 90 days	93	22.0%
More than 90 days	42	9.9%
Reason for the first dental visit in the participant’s lifetime		
Pain	235	55.6%
Routine	30	7.1%
Dental check-up program	50	11.8%
Screening	108	25.5%
Had a dental consultation with any dentist in the previous 12 months, excluding the current appointment?		
No	285	67.4%
Yes	138	32.6%
If yes, what was the main reason for that dental consultation? (*n* = 138)		
Pain	46	33.3%
Treatment	76	55.1%
Routine	15	10.9%
Other	1	0.7%
Main reason for not consulting a dentist in the previous 12 months (*n* = 285)		
Fear	11	3.9%
Consultations are expensive	189	66.3%
Think it is not needed	81	28.4%
Other	4	1.4%
How would you rate your last visit to the dentist? (*n* = 346)		
Very poor	25	7.2%
Poor	43	12.4%
Good	220	63.6%
Excellent	58	16.8%
If you have not been to the dentist in the last 12 months, when was the last time? Mean of years (standard deviation)	6.9	(9.8)
At what age did you first visit the dentist? Mean of years (standard deviation)	12.6	(10.7)
Have you ever had to visit the emergency room for a toothache?		
No	303	71.6%
Yes	120	28.4%
What treatment did you receive in the emergency room for the toothache? (*n* = 120)		
Referred to a stomatologist	14	11.7%
Medication and discharge	106	88.3%

**Table 4:** Association between behavioral and clinical factors and dental pain episodes requiring emergency room services.

	Use of Emergency Room Services (% Yes)		
	*N*	%	*p*-Value *
Smoking habits			
Never smoked	66	20.9%	<0.001
Smokes	41	50.6%	
Former smoker	13	50.0%	
Alcohol consumption habits			
Does not drink	64	22.9%	<0.001
Social drinker	29	37.7%	
Drinks with meals	8	25.8%	
Drinks with meals and outside meals	19	52.8%	
Presence of comorbidities			
No	115	30.0%	0.017
Yes	19	47.5%	
Uses dental prosthesis?			
No	96	25.5%	0.029
Yes	24	51.1%	
Has a “dry mouth”?			
No	67	21.3%	<0.001
Yes	53	48.6%	
Has stomach issues?			
No issues	68	21.4%	<0.001
Has issues	52	49.5%	
Brushes teeth and/or prosthesis daily?			
Does not	21	53.8%	<0.001
Sometimes	52	33.5%	
Once a day	26	27.7%	
Twice a day	18	15.9%	
Three times a day	3	13.6%	
Has a personal toothbrush?			
Manual	103	30.6%	<0.001
Electric	2	8.3%	
Both	4	8.3%	
Does not have	11	78.6%	
Uses toothpaste?			
Does not	25	78.1%	<0.001
Yes	95	24.3%	
Uses dental floss regularly?			
Does not	113	28.8%	0.675
Yes	7	23.3%	
Uses mouthwash or oral elixir as part of daily oral hygiene?			
No	104	30.6%	0.049
Yes	16	19.3%	
Had a dental appointment in the last 12 months?			
No	96	33.7%	0.037
Yes	24	17.4%	

**Note:** * Fisher’s exact test. The presence of comorbidities for dental practice was determined through medication for heart disease, diabetes, hypertension, depression, osteoporosis, epilepsy, renal insufficiency, schizophrenia, and thyroid conditions.

**Table 5:** Descriptive statistics of the components of the DMFT index.

	Median	Min	Max	P25	P75	*p*-Value *
DMFT permanent teeth	5	0	32	0	15	<0.001
DMFT deciduous teeth	0	0	18	0	0	<0.001
Decayed teeth	2	0	32	0	6	<0.001
Missing teeth	0	0	32	0	6	<0.001
Filled teeth	0	0	11	0	1	<0.001

* Shapiro–Wilk Test.

**Table 6:** Components of the DMFT index and age at first visit to the dentist by dental pain episode requiring emergency room services.

	Dental Pain Episode with Emergency Room Use						
	No			Yes			*p*-Value *
	Median	P25	P75	Median	P25	P75	
DMFT permanent teeth	1	0	12	14	7.25	21	<0.001
DMFT deciduous teeth	0	0	0	0	0	0	<0.001
Decayed teeth	1	0	5	4	1	9	<0.001
Missing teeth	0	0	3	6	0	12	<0.001
Filled teeth	0	0	1	0	0	1	0.187
Age at first visit to the dentist	6	4	15	17	10	20	<0.001

* Mann–Whitney U Test.

**Table 7:** Logistic regression analysis of factors associated with emergency room utilization due to dental pain *n* = 120.

Variable	Category/Unit	aOR	95% CI	*p*-Value *
DMFT index for permanent teeth	Per one-point increase	1.071	1.036–1.107	<0.001
Current smoking	Yes vs. No	2.466	1.350–4.506	0.003
Alcohol consumption	Yes vs. No	0.583	0.308–1.106	0.099
Presence of comorbidities	Yes vs. No	0.703	0.369–1.341	0.285
Age	Per one-year increase	1.014	0.998–1.031	0.087

Note: * Emergency room utilization due to dental pain was included as the dependent binary variable. The model was adjusted for age. Results are expressed as adjusted odds ratios (aORs) with 95% confidence intervals (95% CIs). aOR, adjusted odds ratio; CI, confidence interval; DMFT, Decayed, Missing, and Filled Teeth index. A *p*-value < 0.05 was considered statistically significant.

## Data Availability

The data presented in this study are available on request from the corresponding author. The data are not publicly available due to privacy and ethical restrictions.

## Supplementary Materials

The following supporting information can be downloaded at: https://www.mdpi.com/article/10.3390/dj14070411/s1, File S1: Questionnaire. Table S1. Medication, complications and family history. Table S2. Health behaviors and risk factors. Table S3. Use of dental prostheses. Table S4. Association between behavioral and clinical factors and dental pain episodes requiring emergency room services: sensitivity analysis restricted to adult participants (aged ≥ 18 years).
