# Supplementary material for: The Impact of Limited Access to Dental Care on Emergency Room Service Utilization: A Study of Primary Healthcare in a Rural Inland Region of Portugal
Source: Dent J (Basel). 2026 Jul 6;14(7):411. doi: 10.3390/dj14070411 (PMC13408959; doi:10.3390/dj14070411)
Supplement: Supplementary file 1 [file dentistry-14-00411-s001.zip › dentistry-4336692-supplementary.pdf]

Supplementary material

Questionnaire

Sociodemographic information

- **Gender:** Male ___ Female ___ Other ___
- **Age/Date of Birth:**
- **Residence:** Urban Area ___ Rural Area ___
- **Resides in an institution:** Yes ___ No ___
- **Educational level:**
    - No education ___
    - Kindergarten ___
    - Primary education (First cycle) ___
    - Lower secondary (Second cycle) ___
    - Upper secondary (Third cycle) ___
    - High school (Secondary education)
    - Bachelor's degree ___
- **Currently employed:** Yes ___ No ___
- **Marital status:** ___
- **Number of people living in your household**: ___

I – General health

1. Are you currently on medication for any of the following conditions?

- Hypertension ___
- Diabetes ___
- Depression ___
- Cancer ___
- High cholesterol ___
- Acute myocardial infarction ___
- Stroke ___
- Chronic kidney disease, cirrhosis, chronic hepatitis ___
- Asthma ___
- Chronic obstructive pulmonary disease ___
- Chronic pain ___

- Osteoporosis ___
- Rheumatoid arthritis ___
- Peptic ulcer (gastric or duodenal) ___
- Allergies ___
- HIV ___
- Other __________

2. Are you allergic to any medication?

Yes ___ No ___

3. Do you heal well?

Yes ___ No ___

4. Do you have any stomach problems?

Yes ___ No ___

5. Have you ever had surgery?

Yes ___ No ___

If yes, what kind? ______

6. Do you have a history of cancer in your immediate family?

Yes ___ No ___

If yes, which type of cancer? ________ Relative ________

7. Do you smoke?

Yes ___ No ___

If yes, how many cigarettes per day? ___ Since what age? ___

8. Alcohol consumption?

Yes ___ No ___

If yes:

- Socially ___
- During meals ___
- Both during and outside meals ___
- Wine ___
- Beer ___
- Spirits ___

9. Do you practice any sports?

Yes ___ No ___

If yes, how often?

- Once a week ___
- More than once a week ___

10. Do you have recent lab test results?

Yes ___ No ___

If yes, when? ___

II – Oral health

Reason for consultation in primary healthcare

- Pain ___
- Routine ___
- Lesion that does not disappear ___
- To place prosthetics/braces ___
- Did not know the appointment was scheduled ___

Dental Chart

		55	54	53	52	51	61	62	63	64	65		
17	16	15	14	13	12	11	21	22	23	24	25	26	27
47	46	45	44	43	42	41	31	32	33	34	35	36	37
		85	84	83	82	81	71	72	73	74	75		

Legend: Letters for identifying the primary dentition and numbers for permanent dentition.

- A/O – Healthy
- C/2 – Filled with cavity
- E/4 – Lost due to cavity
- B/1 – Cavitated
- D/3 – Filled without cavity
- 5 – Lost due to other reasons
- F/6 – Sealed
- G/7 – Implant or prosthesis
- 8 – Not erupted
- 9 – Excluded
- T – Fractured

Number of decayed teeth	Number of filled teeth	Number of lost teeth	Number of sealed teeth

1. Do you brush your teeth and/or clean your prosthesis daily?

No ___ Yes ___

How many times a day? ___

2. Do you have a personal toothbrush?

No ___ Yes ___

If yes:

- Manual ___
- Electric ___

3. Do you use toothpaste?

No ___ Yes ___

4. Do you use dental floss?

No ___ Yes___ Sometimes ___ Yes, every day ___

5. At what age did you first visit the dentist?

_____ years

Why did you visit?

- Check-up ___
- Toothache ___
- Cleaning ___
- Prosthesis ___
- Dentist-cheque program ___

6. Have you seen a dentist in the last 12 months?

No ___ Yes ___

Why did you go?

- Check-up ___
- Toothache ___
- Cleaning ___
- Prosthesis ___
- Dentist-cheque program ___

7. If you haven't seen a dentist in the last 12 months, when was your last visit?

______ months ago.

8. What is the main reason you did not visit a dentist?

- Consultations are expensive ___
- Cannot find a specialist dentist ___
- Refused dental treatment ___
- Do not feel the need for dental care ___
- Difficult to travel ___
- I regularly see the dentist ___

9. How would you rate your last visit to the dentist?

- Excellent ___
- Good ___
- Poor ___
- Terrible ___

10. Do you experience "dry mouth"?

No ___ Yes ___

11. If yes, do you try to compensate by drinking more water?

No ___ Yes ___

12. Do you grind your teeth?

No ___ Yes ___

13. Do you frequently bite your lips or cheeks?

No ___ Yes ___

14. Have you ever had to visit the emergency services for tooth pain?

No ___ Yes ___

- If yes, what happened?
- Stayed hospitalized ___
- Treated and discharged ___
- Referred to a dental specialist ___

15. How long did you wait for your consultation in primary healthcare?

- 1 Month ___
- 2 to 3 months ___
- More than 3 months ___

III – Dental prosthetics (if applicable)

1. Do you use dental prosthetics?

No ___ Yes ___

If you answered “No,” the questionnaire ends here.

Thank you for your cooperation.

2. When do you use the prosthesis?

- Always ___
- Sometimes ___
- Only during meals ___

3. Do you feel better since you got your current dental prosthesis?

No ___ Yes ___ I don’t know ___

4. Do you feel discomfort or pain when using the prosthesis?

No ___ Yes ___

5. Can you eat normally with the prosthesis?

- Always ___
- Sometimes ___
- Rarely ___
- Never ___

6. Are you satisfied with your prosthesis?

No ___ Yes ___

7. Do you remove the prosthesis to sleep?

- Always ___
- Sometimes ___
- Rarely ___
- Never ___

8. Do you clean your prosthesis?

- Every day ___
- Sometimes ___
- Rarely, once a week ___
- Never ___

8.1. If you clean it every day, how many times per day do you clean your prosthesis?
__________ times.

9. How long have you been using a dental prosthesis?
_______ years.

10. How long have you had your current dental prosthesis?
_______ years.

Thank you for your cooperation.

Table S1. Medication, complications and family history.

	n	%
**Uses medication for any reason** (*n* = 423)		
No	208	49.2
Yes	215	50.8
**Reason for medication** (*n* = 215)		
Polymedicated	143	66.5
Hyperactivity	13	6.0
Oral contraceptive	12	5.6
Diabetes	9	4.2
Asthma	7	3.3
Osteoporosis	5	2.3
Heart conditions	4	1.9
Depression	4	1.9
Kidney insufficiency	4	1.9
Schizophrenia	4	1.9
Hypertension	3	1.4
Epilepsy	3	1.4
Pain	2	0.9
Thyroid conditions	2	0.9
**Allergy to any medication?** (*n* = 423)		
No allergies	369	87.2
Medication allergies	36	8.5
Unknown	18	4.3
**Which medication causes allergies?** (*n* = 36)		
Penicillin	28	77.8
Aspirin	6	16.7
Other	2	5.6
**Heal well?** (*n* = 423)		
Does not heal well	23	5.4
Heals well	400	94.6
**Has stomach problems?** (*n* = 423)		
No problems	318	75.2
Has problems	105	24.8
**Takes medication for stomach problems** (*n* = 105)		
No	32	30.5
Yes	27	25.7
Only occasionally	46	43.8
**Has undergone surgery?** (*n* = 423)		
No surgeries	255	60.3
Has undergone surgery	168	39.7
**Which surgeries?** (*n* = 168)		
Various	54	32.1
Otorhinolaryngological	25	14.9
Orthopedic	22	13.1
Hernia	22	13.1
Appendectomy	10	6.0
Oncological surgery	9	5.4
Cardiac	8	4.8
Dental surgery	6	3.6
Kidney	6	3.6
Circumcision	6	3.6
**Cancer in direct family members?** (*n* = 423)		
No history	287	67.8
Has a history	136	32.2
**Cancer history (*n* = 136)**		
Gastric cancer	70	51.5
Breast cancer	28	20.6
Lung cancer	13	9.6
Lymphoma	11	8.1
Prostate cancer	8	5.9
Throat cancer	4	2.9
Skin cancer	2	1.5
**Family member** (*n* = 136)		
Mother	41	30.1
Father	38	27.9

Paternal grandfather	16	11.8
Siblings	14	10.3
Maternal grandfather	11	8.1
Maternal grandmother	8	5.9
Paternal grandmother	5	3.7
Self	3	2.2

Table S2. Health behaviors and risk factors.

	n	%
**Engages in sports? (*n* = 423)**		
Does not engage	343	81.1
Once a week	37	8.7
More than once a week	33	7.8
30-minute walks daily	10	2.4
**Has recent laboratory tests? (*n* = 423)**		
Never had tests	106	25.1
More than 1 year ago	107	25.3
More than 3 months ago	89	21.0
Approximately 3 months ago	39	9.2
Last month	82	19.4
**Alcohol consumption habits (*n* = 423)**		
No	279	66.0
Socially	77	18.2
During meals	31	7.3
During meals and outside meals	36	8.5
**If yes, what do they drink? (*n* = 144)**		
Wine	65	45.1
Beer	19	13.2
Wine and beer	3	2.1
Spirits	20	13.9
All the above	35	24.3
**Smoking habits (*n* = 423)**		
Never smoked	316	74.7
Smokes	81	19.1
Former smoker	26	6.1
**If yes, how many per day:** Mean = 25.2 (SD = 18.5); Min. = 3, Max. = 80		
**Age when smoking started:** Mean = 14.6 (SD = 18.2); Min. = 7, Max. = 30		
**Age when quit smoking:** Mean = 46.9 (SD = 12.3); Min. = 26, Max. = 74		
**Years as a smoker:** Mean = 31.0 (SD = 10.2); Min. = 9, Max. = 44		

Table S3. Use of dental prostheses.

	n	%
**Uses dental prostheses? (*n* = 423)**		
No	376	88.9
Yes	47	11.1
**Type of dental prostheses (*n* = 47)**		
Fixed total	3	6.4
Partial acrylic removable	38	80.9
Total acrylic removable	6	12.8
**When do you use the dental prosthesis? (*n* = 47)**		
Sometimes	14	29.8
Always	33	70.2
**Do you feel better since you got your current dental prosthesis? (*n* = 47)**		
No	10	21.3
Yes	35	74.5
Sometimes	2	4.3
**Do you experience any discomfort or pain while using the dental prosthesis? (*n* = 47)**		
No	25	53.2
Yes	15	31.9
Sometimes	7	14.9
**Can you eat normally with the dental prosthesis? (*n* = 47)**		
No	13	27.7
Yes	24	51.1
Sometimes	10	21.3
**Are you satisfied with the dental prosthesis? (*n* = 47)**		
Not satisfied	21	44.7
Satisfied	12	25.5
Completely satisfied	14	29.8
**Do you usually remove the dental prosthesis at night? (*n* = 47)**		
Yes	16	34.0
Sometimes	6	12.8
Never	22	46.8
It is fixed, does not come out	3	6.4
**Do you usually clean your dental prosthesis? (*n* = 47)**		
No	2	4.3
Yes	21	44.7
Sometimes	24	51.1
**How long have you been using a dental prosthesis? (*n* = 47)**		
1 year	4	8.5
2 to 5 years	10	21.3
5 to 10 years	12	25.5
10 to 20 years	10	21.3
More than 20 years	11	23.4
**How long have you had the current dental prosthesis?** Mean = 8.04 (SD = 6.3); Min. = 1; Max. = 20		

Table S4. Association between behavioral and clinical factors and dental pain episodes requiring emergency room services: sensitivity analysis restricted to adult participants (aged ≥ 18 years).

	Use of emergency room services(% Yes)		
	n	%	*p*-Value *
Smoking habits			
Never smoked	42	17.4%	0.198
Smokes	38	15.7%	
Former smoker	13	5.5%	
Alcohol consumption habits			
Does not drink	36	14.9%	0.056
Social drinker	26	10.7%	
Drinks with meals	8	3.3%	
Drinks with meals and outside meals	19	7.9%	
Presence of comorbidities			
No	26	10.7%	0.434
Yes	78	32.2%	
Uses dental prosthesis?			
No	80	33.1%	0.139
Yes	24	9.9%	
Has a “dry mouth”?			
No	51	21.1%	0.027
Yes	53	21.9%	
Has stomach issues?			
No issues	52	21.5%	0.047
Has issues	52	21.5%	
Brushes teeth and/or prosthesis daily?			
Does not	17	7%	0.064
Sometimes	45	18.6%	
Once a day	21	8.7%	
Twice a day	18	7.4%	
Three times a day	3	1.2%	
Has a personal toothbrush?			
Manual	87	36%	0.012
Electric	2	0.8%	
Both	4	1.7%	
Does not have	11	4.5%	
Uses toothpaste?			
Does not	21	8.7%	<0.001
Yes	83	34.3%	
Uses dental floss regularly?			
Does not	97	40.1%	0.145
Yes	7	2.9%	
Uses mouthwash or oral elixir as part of daily oral hygiene?			
No	88	36.4%	<0.001
Yes	16	6.6%	
Had a dental appointment in the last 12 months?			

No	84	34.7%	<0.001
Yes	20	8.3%	
